# Introduction of a Semi-Quantitative Image-Based Analysis Tool for CBCT-Based Evaluation of Bone Regeneration in Tooth Extraction Sockets

**DOI:** 10.3390/bioengineering12030301

**Published:** 2025-03-16

**Authors:** Anja Heselich, Pauline Neff, Joanna Śmieszek-Wilczewska, Robert Sader, Shahram Ghanaati

**Affiliations:** 1 FORM-Lab, Department for Oral, Cranio-Maxillofacial and Facial Plastic Surgery, Medical Center of the Goethe University Frankfurt, Goethe University, 60590 Frankfurt am Main, Germany; 2 Dentist Clinic, Lelewela 1/1, 44-100 Gliwice, Poland

**Keywords:** socket preservation, bone regeneration, PRF, RCT, radiological evaluation

## Abstract

After tooth extraction, resorptive changes in extraction sockets and the adjacent alveolar ridge can affect subsequent tooth replacement and implantation. Several surgical concepts, including the application of autologous blood concentrate platelet-rich fibrin (PRF), aim to reduce these changes. While PRF’s wound-healing and pain-relieving effects are well-documented, its impact on bone regeneration is less clear due to varying PRF protocols and measurement methods for bone regeneration. This study aimed to develop a precise, easy-to-use non-invasive radiological evaluation method that examines the entire extraction socket to assess bone regeneration using CBCT data from clinical trials. The method, based on the freely available Image J-based software “Fiji”, proved to be precise, reproducible, and transferable. As limitation remains the time requirement and its exclusive focus on radiological bone regeneration. Nevertheless, the method presented here is more precise than the ones currently described in the literature, as it evaluates the entire socket rather than partial areas. The application of the novel method to measure mineralized socket volume and radiological bone density of newly formed bone in a randomized, controlled clinical trial assessing solid PRF for socket preservation in premolar and molar sockets showed only slight, statistically non-significant trends toward better regeneration in the PRF group compared to natural healing.

## 1. Introduction

Despite advancements in modern dentistry, millions of teeth are extracted annually, primarily due to caries, endo-periodontal diseases, or trauma. Following a tooth extraction, the alveolar socket undergoes a complex healing process consisting of three phases. The current understanding is that there is the inflammatory phase, the proliferative phase, and the regenerative phase [1,2]. This process begins with the formation of a blood clot and an inflammatory response, followed by the activation of growth factors and the eventual regeneration of bone [2]. The entire process can take up to 12 weeks, and is influenced by factors such as age and overall health status [3,4]. Recent findings suggest that the healing process in the alveolar socket may not follow the same principles as those observed in long bones. Instead of a primary regeneration originating from the lateral aspects of the defect, the process appears to be driven by the collapse of the alveolar walls towards the center. This inward movement of the socket walls leads to bone apposition along the socket margins, but does not necessarily contribute to complete regeneration in the defect center. While crestal closure of the wound occurs, cavitation remains in the apical region in many cases [5]. This is supported by the fact that tooth removal often results in changes to the alveolar ridge, including vertical and horizontal bone loss [6,7], and especially affects the vestibular bone walls [3,7,8,9]. Contributing factors include the lack of mechanical load and the structural characteristics of bundle bone [10,11]. Early interventions are, therefore, critical to minimizing bone loss and preserving the foundation for future prosthetic restorations.

A common and widely used approach to preserving bone and promoting bone regeneration is known as socket or ridge preservation. Socket preservation primarily aims to maintain intact extraction sockets without defects, particularly with a preserved vestibular wall, often classified as type 1 sockets. In contrast, ridge preservation is used when one or more alveolar walls are partially or completely missing, typically referred to as type 2 sockets. In the literature, these terms are often used interchangeably [12]. Various reviews have summarized and compared different techniques and materials for socket and ridge preservation, underscoring their importance as standard therapeutic methods for supporting bone regeneration [12,13,14,15]. Innovative approaches, such as the sole or additional application of the autologous blood concentrate platelet-rich fibrin (PRF), have shown great promise in promoting wound healing and bone regeneration. Studies indicate that PRF, derived from the patient’s own blood and rich in growth factors, can accelerate regeneration without the need for anticoagulants. The effects of PRF on bone healing and alveolar ridge preservation have been examined in numerous studies, comparing various preparation protocols and control groups undergoing spontaneous healing [16,17,18,19,20,21,22,23].

The assessment of bone changes following tooth extraction involves both clinical and imaging techniques. In some published trials, measurements of alveolar ridge width and height were performed using calipers or compasses immediately after extraction and at subsequent intervals [19,20]. Other studies employed customized stents to enable reproducible measurements on dental casts using periodontal probes [16,21]. Two-dimensional radiographs and three-dimensional cone beam computed tomography (CBCT) have played a pivotal role in evaluating bone regeneration in several studies. The utilized techniques included the use of reconstruction software to compare bone volumes three months post-extraction [17] and measurements of vertical and horizontal bone loss through sagittal and axial slices of CBCT images [17,22,23,24,25,26,27]. Additionally, bone density was assessed using gray value analysis from CBCT sections taken from the center of the socket [25,28] or through two-dimensional radiographs [19,28].

A wide range of studies highlight the potential of socket or ridge preservation techniques to positively impact bone regeneration and alveolar ridge stability following tooth extraction. However, significant challenges remain in identifying the optimal biomaterials, PRF applications, or regeneration timelines that yield the best outcomes for ridge preservation and bone regeneration. Although various clinical and radiological methods have been used to assess qualitative and quantitative bone regeneration, which already provided detailed insights into changes in bone dimensions and density following socket healing, the heterogeneity in these approaches makes the results difficult to compare. Furthermore, subjective and often manual measurement techniques limit the reproducibility and reliability of the findings.

The aim of this study was therefore to develop a semi-automated imaging-based method to enable reproducible, semi-quantitative assessments of bone regeneration in tooth extraction sockets using CBCT data, and further to apply this technique for the evaluation of a randomized controlled clinical trial dealing with the suitability of PRF as a sole filling biomaterial for the preservation of tooth extraction sockets.

## 2. Materials and Methods

### 2.1. Software and Data Format

The imaging software Fiji (Fiji Is Just ImageJ, v2.15.0) served as the foundation for the image-based evaluation of bone regeneration. Fiji is built on ImageJ2, an advanced version of the scientific and medical image analysis software ImageJ provided by the National Institutes of Health (NIH, Bethesda, MD, USA; imagej.nih.gov, accessed on 14 May 2024) [29]. All processing and analyzing tools used and implemented in the analysis macro are included in Fiji.

The 3D cone beam computed tomography (CBCT) images used for analysis had been recorded and analyzed in DICOM format.

### 2.2. Clinical Trial Study Design

#### 2.2.1. Study Design

This prospective, parallel-arm randomized controlled trial (RCT) was conducted at the Medical University of Silesia, Katowice, Poland, from January 2018 to May 2022. The study followed the Declaration of Helsinki and national regulations in Poland and Germany for human studies.

Surgical interventions and follow-ups, performed by SG and JS, were approved by the Silesian Medical Council (#30/2017). After tooth extraction and either PRF treatment or no treatment, primary closure was neither achieved nor intended. The study aimed to assess secondary intention wound healing in terms of bone and soft tissue regeneration (published in [30]). All participants provided written informed consent after being informed of the study’s procedures and objectives.

#### 2.2.2. Inclusion and Exclusion Criteria

Patients (≥18 years) requiring premolar or molar extractions (excluding 3rd molars) and planned for dental implant therapy were recruited. Complete medical and dental histories were obtained. Exclusion criteria included lack of written consent, pregnancy, decompensated metabolic disease, untreated periodontal disease, periapical lesions, bisphosphonate-related MRONJ risk, poor oral hygiene, or inability to follow study instructions. Cases with root fractures, residual fragment risk, or pre-existing alveolar bone defects were not included.

#### 2.2.3. Sample Size Calculation

In consultation with our statistical institute, the sample size for the overall clinical trial was calculated based on established criteria [31]. To achieve 80% power at a 5% significance level, a minimum of 13 extractions per group was required. Considering an estimated 20% drop-out rate, the final sample size was set at 16 extractions per group.

#### 2.2.4. Randomization

Patients were randomly assigned to either the control or PRF treatment group. Based on the sample size calculation, computer-generated randomization (GraphPad Software, version 10.3.1, LLC, Boston, MA, USA, www.graphpad.com) was performed by a study member uninvolved in treatment. Each patient was allocated to a sealed randomization letter which was opened by the surgeon immediately before extraction.

#### 2.2.5. Follow-Up and Outcome Measures

Tooth extraction and treatment were assessed for bone condition and regeneration at day 0 and day 90 post-extraction. The primary outcome was bone volume gain three months after extraction.

### 2.3. PRF Preparation

Autologous platelet-rich fibrin (PRF) was prepared, as previously described [32,33]. In the PRF treatment group, peripheral blood was drawn into 10 mL additive-free PRF vacuum tubes (Process for PRF, Nice, France) for centrifugation and application at premolar and molar extraction sites. One tube per root was collected, determined by pre-extraction X-ray and randomization. Samples were centrifuged in a PRF-Duo Quattro device (Process for PRF, Nice, France) within 3 min of collection at 1200 rpm (177× *g*) for 8 min. If needed, a sodium chloride-filled tube was used for balance. The resulting PRF clot was extracted with sterile tweezers, residual red blood cells were removed, and the clot was transferred to a PRF processing box for compression into a cylindrical PRF plug using gravitational force. The plug was applied to the extraction socket within 20 min of processing.

### 2.4. Surgical Procedures

#### 2.4.1. Tooth Extraction and Socket Preservation

Extractions were performed under local anesthesia using a minimally traumatic technique without vertical releasing incisions. To minimize root and bone fracture risk, molars were sectioned via piezo technique. Sockets were inspected, curetted, and rinsed with sterile 0.9% saline. In the PRF group, sockets were filled with solid PRF and sutured tension-free with non-resorbable horizontal mattress sutures. Control sockets remained untreated but received identical suturing.

#### 2.4.2. Follow-Up and Radiological Evaluation

In addition to clinical evaluation and photo documentation, CBCT imaging was performed post-extraction and before implantation at three months. Bone regeneration was assessed based on these CBCT scans. CBCTs were subjected to a qualitative analysis prior to evaluation, and CBCTs with optical artifacts, such as overexposed areas from adjacent prosthetic restorations or incomplete alveoli, were excluded to ensure a reliable assessment. Evaluation of the mineralized alveolar volume and radiologically assessed bone density could finally be performed in n = 7 molar and n = 5 premolar sockets in the solid PRF group, and n = 12 molar and n = 8 premolar sockets the control group.

## 3. Results

### 3.1. Method Development

A new image analytical evaluation method for bone regeneration in extraction sockets was developed using a semi-automated step-by-step processing approach of CBCT-images. The two endpoints to be evaluated are mineralized bone volume, representing the (re)generation of new bone tissue, and bone density, representing the grade of mineralization within the extraction alveola. For this DICOM raw data of the CBCTs of two time points (A: post tooth extraction, and B: after a defined regeneration time, e.g., 3 months) were processed as follows.

The evaluation process is divided into the following:

(i) Pre-processing of CBCT images;
(ii) Defining region of interest and measurement;
(iii) Parameter evaluation based on achieved raw data.

(i) Preparation and Processing of CBCTs for Measurements

First, CBCTs of both time points were cropped to the region of interest including the area of the extraction alveola and optimized for smoother processing. For this, the compressed DICOM raw data were loaded into Fiji via the *Bio-Formats Importer* plugin, the grayscale range was homogenized to 8-bit, with the DICOM data then displayed in 256 grayscale levels, and saved in TIFF format for smoother processing. The voxel size of the CBCTs was checked and in case of compression re-adjusted to original dimensions using the properties function.

Then, the *slice keeper* tool was used to remove unnecessary apical and coronal regions containing the mandibular canal, opposing jaw, or parts of the maxillary sinus in the CBCTs, while retaining slices that included parts of the empty socket and adjacent teeth.

**Figure 1:**
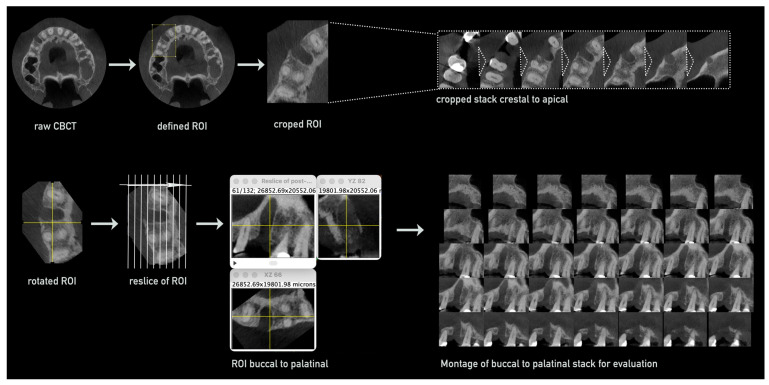
Semi-automated CBCT processing for volume and density evaluation. The raw CBCT data were imported, followed by the definition of the region of interest (ROI) and cropping to isolate the area including the treated socket. The resulting stack, encompassing the entire region from the crestal to the apical aspect, was resliced from buccal to palatal/lingual. For the final evaluation, a montage of the resliced buccal-to-palatal/lingual stack was created.

Further reduction to the region of interest was achieved by defining a rectangular area around the socket and its adjacent teeth in the axial slice using the *rectangular* tool and cropping the CBCT stack in all dimensions—apical–coronal, vestibular–oral, and mesial–distal—using the *crop* function (Figure 1, upper row).

The cropped stack was then rotated using the *rotate* function to align the socket and adjacent teeth along a vertical line. Next, the stack was re-sliced in a vestibular to oral orientation using the *reslice* function, creating a new stack with slices perpendicular to the original axial slices. Further reduction of the stack was performed to retain only slices containing parts of the socket, with more distal slices removed using the *slice keeper* tool. This resulted in a stack composed of approximately 40–70 slices, depending on the vestibular–oral width of the socket (Figure 1, lower row).

For an optimized measurement process, every other slice was removed from the stack using the *slice keeper* function with Increment: 2, leaving approximately 20–35 slices. A montage was created from this stack, showing all remaining slices of the socket from vestibular to oral (Figure 1).

For consistent processing of the second CBCT taken after the regeneration period, the rotated and cropped stack of the first CBCT (post extraction) was used as a reference. After the reslicing of the original CBCT, the *slice keeper* and *rotation* tools were applied to align the stacks from both CBCTs, ensuring identical slices with minimal rotation discrepancies for the following measurements.

(ii) Defining Region of Interest and Measurement within Montages

Within the created montage of the processed CBCT stacks, the regions of interest—the extraction alveolus in the first CBCT and the regenerated bone tissue in the second CBCT—were marked and measured as follows: to define relevant structures for orientation and measurement, the Polygon tool was used to mark these areas. To correctly transfer the defined region of the original alveolus to the second CBCT, a reference, typically a tooth or characteristic ridge contour, was first marked in the first CBCT, starting with the most vestibular slice. The resulting ROI (region of interest) was then saved in the *ROI manager*. For each slice, the region of the alveolus was marked, the corresponding ROI was added to the *ROI manager*, and the area of the ROI was measured using the *measure* function.

This process was repeated for every slice in the montage. Resulting parameters, such as the area of the marked alveolar sections and the average grayscale values, were stored for later calculations. Additionally, small areas within the spongy bone region in each slice were defined to determine the reference grayscale value of the endogenous bone. These areas were also measured and stored.

The montage from the second CBCT was then opened, and the marking and measurement procedure was repeated, with careful attention given to transferring the reference from the first CBCT to the second, ensuring proper alignment and accuracy. The marked alveolar areas were then measured for mineralized portions only, using the *measure* function again. The recorded parameters included the area of the mineralized alveolar portion and the average grayscale values.

(iii) Parameter Evaluation Based on Achieved Raw Data

The volume of the empty socket immediately after tooth extraction was calculated by summing all measured alveolar areas and multiplying them by the slice thickness. The latter was double the voxel size or depth, as every other slice had been removed during the processing of the CBCT for evaluation. Similarly, the areas of the mineralized alveolar regions from the second CBCT, generated from the DICOM data after bone regeneration, were processed. To compare the regeneration values between samples and groups, independent of the individual size of each socket, the relative proportion of the regenerated mineralized volume in relation to the original alveolar volume was calculated by dividing the volume of the mineralized alveolar regions by the volume of the socket immediately after extraction.

$$Mineralized\;socket\;volume\;(\%)=\frac{\sum mineralized\;area\;post\;regeneration\;\times slice\;thickness}{\sum area\;of\;post\;extraction\;alveola\times slice\;thickness}\times 100$$

### 3.2. Radiological Bone Density Evaluation

**Figure 2:**
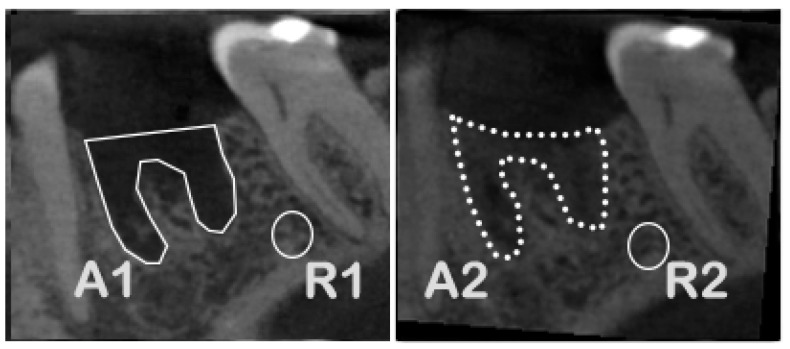
Radiological bone density assessment. **Left**: cross-section of extraction socket post-extraction, **Right**: cross-section of extraction socket after regeneration. Defined are the region of the extraction socket (A1 and A2) and the bone reference for measurements (R1 and R2).

Since CBCT imaging typically lacks standardized calibration settings, making density measurements using Hounsfield values impractical, an alternative approach was needed to calculate the density changes within the area of interest. Therefore, the second parameter of bone regeneration, radiological bone density, was evaluated through grayscale measurements of newly formed bone within the socket, compared to the surrounding spongy alveolar bone. Due to slight variations in CBCT settings, average grayscale values differed, making a direct comparison of grayscale values not possible. To eliminate errors from these differences, small ellipses of uniform size were placed in the spongy bone region as reference points (see Figure 2).

**Figure 3:**
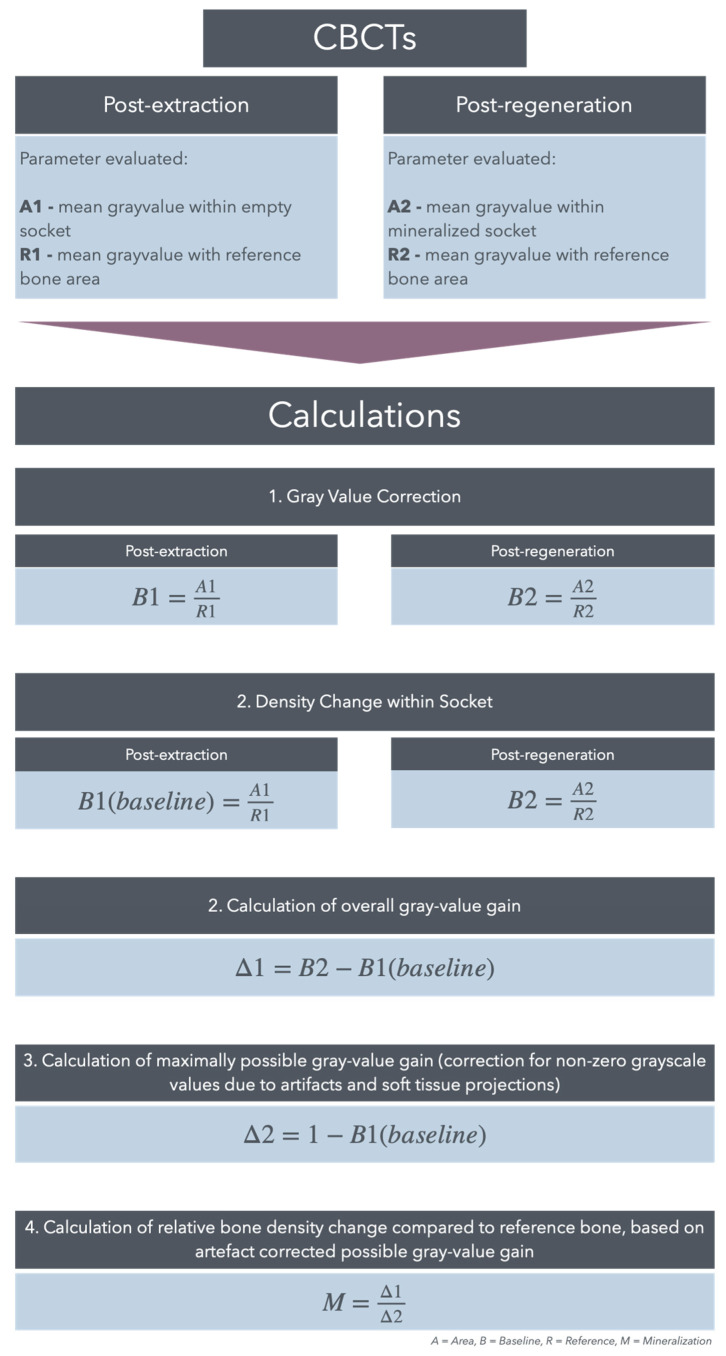
Radiological bone density assessment. Calculation of gray values and alveolar mineralization relative to reference bone.

The average grayscale values within these reference regions, as well as mean grayscale values for the empty alveolus immediately after extraction and the newly formed bone after regeneration, were measured in parallel to the volume evaluation in the CBCTs at both time points. The radiological bone density of the newly formed bone was calculated by comparing values within the extraction alveola to the reference bone. Since the empty alveolus showed non-zero grayscale values due to artifacts and soft tissue projections, these were corrected, as shown in the process flow chart (Figure 3), which allowed for an accurate assessment of mineralization increase in the alveolus relative to the reference bone.

### 3.3. Application of the Novel Method in the Clinical Trial

#### 3.3.1. Quality Assessment of Datasets for Evaluation

To assess bone regeneration after tooth extraction, CBCT datasets were reviewed for completeness and compatibility with the evaluation method. Raw data from 16 extraction sockets per group and tooth type were available. A total of 19 molar and 13 premolar alveoli (control group: 12 molars, 8 premolars; solid PRF group: 7 molars, 5 premolars) were suitable for evaluation. Exclusions were made for incomplete alveolar representation, deviations from the planned three-month period, small sockets (likely post-hemi-section), insufficient image quality, or differing voxel dimensions between CBCTs.

#### 3.3.2. Evaluation of Mineralized Bone Volume

The proportion of mineralized volume after three months relative to the total alveolar volume post-extraction was determined, and a comparison of the study groups (solid PRF and control) was performed for both premolars and molars, including all sockets and separately for type 1 and type 2 sockets. Type 1 sockets were defined as sockets with both the vestibular and oral lamellae fully preserved during tooth extraction, while type 2 sockets showed at least one lamella, typically the vestibular one, as not fully preserved. The classification into type 1 or type 2 sockets was made during the initial evaluation of the CBCTs in axial slices using Fiji.

##### Mineralized Bone Volume in Molars

**Figure 4:**
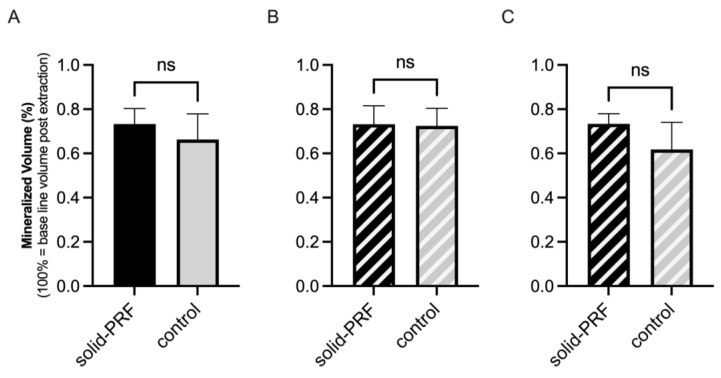
Mineralized bone volume (molars). Evaluation of the mineralized proportion of molar sockets three months post-extraction. Comparison of solid PRF and control group, including all molar sockets (**A**), and further subdivision into type 1 sockets (**B**) and type 2 sockets (**C**). No significant differences were observed between the groups, regardless of socket type. Data are presented as mean ± SD (ns = no statistically significant differences).

For molar sockets, the average mineralized alveolar proportion was 73.3% ± 7.0% in the solid PRF group (nPRF = 7) and 66.3% ± 11.6% in the control group (nCtrl = 12) (Figure 4A). After classifying the sockets into type 1 and type 2, the average mineralized alveolar proportion for type 1 sockets (nPRF = 5, nCtrl = 5) was 73.2% ± 8.3% in the solid PRF group and 72.5% ± 7.9% in the control group (Figure 4B). For type 2 sockets (nPRF = 2, nCtrl = 7), the average mineralized alveolar proportion was 73.4% ± 4.5% in the solid PRF group and 61.8% ± 12.2% in the control group (Figure 4C). A tendency for better volume regeneration was observed for the solid PRF group for total analysis and for type 2 sockets (Figure 4A,C); however, the differences were not statistically significant.

##### Mineralized Bone Volume in Premolars

**Figure 5:**
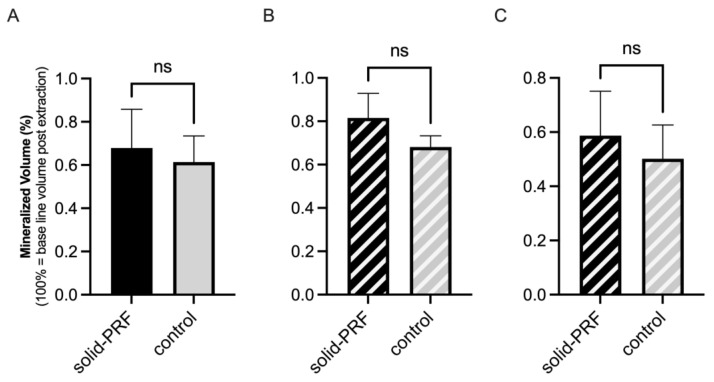
Mineralized bone volume (premolars). Evaluation of the mineralized proportion of premolar sockets three months post-extraction. Comparison of solid PRF and control group, including all premolar sockets (**A**), and further subdivision into type 1 sockets (**B**) and type 2 sockets (**C**). No significant differences were observed between the groups, regardless of socket type. Data are presented as mean ± SD (ns = no statistically significant differences).

For premolar sockets, the average mineralized alveolar proportion related to total socket volume post-extraction for all sockets was 67.5% ± 18.0% in the solid PRF group (nPRF = 5) and 61.4% ± 12.1% in the control group (nCtrl = 8) (Figure 5A). After classifying the sockets into type 1 and type 2, the average mineralized alveolar proportion for type 1 sockets (nPRF = 2, nCtrl = 5) was 81.6% ± 11.3% in the solid PRF group and 68.1% ± 5.2% in the control group (Figure 5B). For type 2 sockets (nPRF = 3, nCtrl = 3), the average mineralized alveolar proportion was 58.7% ± 16.4% in the solid PRF group and 50.2% ± 12.5% in the control group (Figure 5C). In premolar sockets, a tendency for better volume regeneration was observed for the solid PRF group in the total analysis and the sub-analysis (Figure 5); however, the differences were not statistically significant.

#### 3.3.3. Evaluation of Radiological Bone Density Within Former Extraction Sockets

The radiologically assessed bone density of newly formed bone tissue in extraction sockets, compared to the surrounding spongy alveolar bone, was initially determined in all sockets, followed by a subdivision into type 1 and type 2 sockets.

##### Radiological Bone Density in Molars

**Figure 6:**
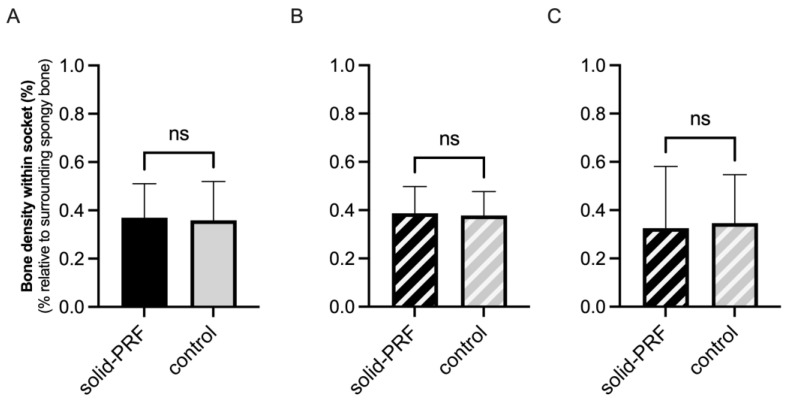
Radiological bone density within former extraction sockets (molars). Evaluation of the average radiological bone density of newly formed bone in molar sockets compared to surrounding spongy alveolar bone three months post-extraction. Comparison of the solid PRF and control groups, including all molar sockets (**A**), and further subdivision into type 1 (**B**) and type 2 sockets (**C**). No statistically significant differences were found between the groups, regardless of socket type. Data are presented as mean ± SD (ns = no statistically significant differences).

For molar sockets, the average radiological bone density, including all sockets, was 36.9% ± 14.1% in the solid PRF group (nPRF = 7) and 35.9% ± 16.1% in the control group (nCtrl = 12), relative to the mean radiological density of the surrounding spongy bone (Figure 6A). Sub-analysis of type 1 and type 2 sockets showed an average bone density for type 1 sockets (nPRF = 5, nCtrl = 5) of 38.7% ± 11.1% in the solid PRF group and 37.8% ± 10.0% in the control group (Figure 6B). For type 2 sockets (nPRF = 2, nCtrl = 7), the average radiological bone density was 32.5% ± 25.6% in the solid PRF group and 34.6% ± 20.1% in the control group (Figure 6C). In molar sockets, no statistically significant differences between the groups could be observed.

##### Radiological Bone Density in Premolars

**Figure 7:**
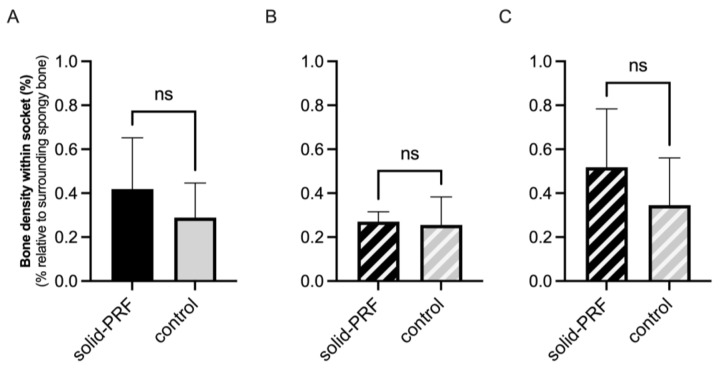
Radiological bone density within former extraction sockets (premolars). Evaluation of the average radiological bone density of newly formed bone in premolar sockets compared to surrounding spongy alveolar bone three months post-extraction. Comparison of the solid PRF and control groups, including all premolar sockets (**A**), and further subdivision into type 1 (**B**) and type 2 sockets (**C**). No statistically significant differences were found between the groups, regardless of socket type. Data are presented as mean ± SD (ns = no statistically significant differences).

The average radiologically determined bone density was evaluated for premolar sockets for all sockets, and was 41.9% ± 23.3% in the solid PRF group (nPRF = 5) and 28.9% ± 15.7% in the control group (nCtrl = 8), relative to the mean radiological density of the surrounding spongy bone (Figure 7A). For type 1 sockets (nPRF = 2, nCtrl = 5), the average radiological bone density was 26.9% ± 4.5% in the solid PRF group and 25.5% ± 12.7% in the control group (Figure 7B). For type 2 sockets (nPRF = 3, nCtrl = 3), the average bone density was 51.8% ± 26.5% in the solid PRF group and 34.5% ± 21.6% in the control group (Figure 7C). In premolar sockets, a tendency for higher radiological bone density was observed for the solid PRF group in the total analysis (Figure 7A) and in the sub-analysis of type 2 sockets (Figure 7C); however, the differences were not statistically significant.

## 4. Discussion

Proficient bone preservation and regeneration in post-tooth extraction sockets is a critical issue in dental medicine, especially when implant-based prosthetics are planned. In addition to the conventional approach of natural healing, a common therapy to achieve this is socket or ridge preservation, which involves the application of biomaterials into the socket [12,13,14,15]. A special interest lies within the application of autologous materials, especially of the application of the autologous blood concentrate platelet-rich fibrin (PRF) to promote the regeneration process. Various types of blood concentrates exist, each with distinct characteristics depending on the applied centrifugation and processing protocols, including differences in leukocyte and platelet content, fibrin structure, and growth factor release. Examples include platelet-rich plasma (PRP), plasma rich in growth factors (PRGF), and platelet-rich fibrin (PRF). Platelet-rich fibrin (PRF), a second-generation blood concentrate, offers the advantage of being produced without anticoagulants or other additives and can be obtained in either liquid or solid form [30]. A variety of autologous blood concentrate variants and corresponding socket or ridge preservation protocols are the focus of numerous clinical trials [16,17,19,20,21,22,23,24,25,26,27]. However, non-invasive (semi-)quantitative evaluation of bone regeneration is a challenging task. Several approaches have been proposed, but they often suffer from low compatibility and difficult reproducibility, making it still challenging to judge the efficacy of autologous blood concentrates for socket/ridge preservation approaches. To address this issue, the aim of this study was to develop a semi-automated, imaging-based method that enables reproducible, semi-quantitative assessments of bone regeneration in tooth extraction sockets using CBCT data. This method aims to establish comparability, allowing for the reproducible, quantitative comparison of different approaches. The newly developed image analysis method was applied to analyze bone regeneration parameters using radiological data from a randomized controlled trial. The aim was to describe bone regeneration and changes in extraction sockets during healing, both qualitatively and quantitatively. Two patient groups were analyzed: one received socket preservation with solid PRF after tooth extraction, while the other served as a control group. Bone regeneration and changes around the socket were evaluated for the entire cohort, as well as by tooth type and socket types 1 and 2.

### 4.1. Development of a Novel Image Analysis-Based Evaluation Method

The evaluation method developed here for the radiological assessment of bone regeneration in extraction sockets distinguishes itself through its comprehensive analysis and precision compared to many approaches described in the literature. However, specific weaknesses also become apparent, which affect the comparability with other methods and study results.

A central advantage of the method developed in this study is the three-dimensional assessment of the entire extraction socket using CBCT data. This contrasts with methods that rely solely on two-dimensional periapical radiographs (PAs), such as those used by Alzahrani et al. [16] and Girish Kumar et al. [19]. The precision of the developed method is enhanced by overcoming the structural superimpositions inherent in two-dimensional imaging. However, compared to literature methods, many of these approaches are considerably more time-efficient, albeit at the expense of accuracy. Methods like those of Castro et al. [18] and Temmerman et al. [26], which use CBCT data, often focus on the center of the socket and disregard the marginal areas. This can lead to an overestimation of bone regeneration. In contrast, despite demanding more time for preparation and evaluation, which can, in some cases, add up to a total of one hour per patient, the method used in this study allows for a systematic and differentiated examination of the entire socket, which is particularly important for analyzing vestibular resorption processes. However, the method places high demands on the quality of CBCT data, as inconsistencies in voxel size between post-extraction and post-regeneration CBCTs can significantly affect measurement accuracy. This highlights a technical sensitivity that is rarely addressed in the literature.

The radiological assessment of the degree of mineralization by analyzing grayscale values in radiographs has been comparatively documented in only a few studies. Methods such as the use of the Hounsfield scale, employed by Anitua et al. [34] and Yewale et al. [22], provide an established metric for assessing bone density. In contrast, the present method requires manual definition of regions of interest (ROIs), making it more labor-intensive and error-prone. Nevertheless, incorporating the surrounding bone as a reference, as performed by Yewale et al. [22], facilitates a better evaluation of relative mineralization increase. A strength of the present method is its comprehensive consideration of the entire socket, including marginal areas, enabling the detection of differences in mineralization between the socket center and the periphery. These aspects are often overlooked in the literature, particularly in two-dimensional approaches where overlapping structures preclude such detailed analysis. However, using relative values for radiological bone density would enhance comparability with other studies.

While commercial software solutions, like MeVisLab or Invivo [17,18], significantly save time through automatic segmentations, the developed method requires labor-intensive manual processing. The time commitment, particularly for precise alignment and processing of CBCT data, is high, increasing the risk of inaccuracies. The literature demonstrates that automated approaches are often sufficiently precise for many applications, although they are limited in terms of transparency and reproducibility. The present study deliberately opted for manual steps to maintain control over data processing and analysis. While this approach enhances precision, it comes at the expense of efficiency. However, by having the method based on the free-access available and widely applied image-analysis platform Fiji (based on NIH’s ImageJ) [29], the application can be easily, and at no cost, established in other research groups as well.

### 4.2. Efficacy of Solid PRF for Bone Regeneration—Mineralized Tissue Volume

The semi-quantitative image analysis was used to assess the volume of newly mineralized tissue in extraction sockets three months after tooth extraction. The proportion of mineralized volume to total socket volume showed no significant difference between the solid PRF and control groups for molars (solid PRF: 73.3% ± 7.0%, control: 66.3% ± 11.6%) or for premolars (solid PRF: 67.5% ± 18.0%, control: 61.4% ± 12.1%), nor when subdividing by socket types 1 and 2. However, a tendency for more mineralized socket volume was observed in the solid PRF group.

Given the variability in the accuracy of radiological methods for assessing the bone-filled portions of sockets and the diverse evaluation approaches used in existing literature, it is important to note that comparing the data obtained with the highly precise analysis method used in this study to those used in the literature may be challenging.

Castro et al. found significantly higher socket filling with regenerated bone in the L-PRF (85.2% ± 22.9%) and A-PRF+ (83.8% ± 18.4%) groups compared to the control group (67.9% ± 19.2%) [18]. In contrast, only trends, but no significant difference towards higher mineralization in the PRF group, were observed in this study. Overall, the values for socket filling were lower compared to Castro et al. Canellas et al. reported more newly formed bone volume in the PRF group [17], while Girish Kumar et al. found no significant differences between groups for socket filling with regenerated bone after six months (control group: 74.3% ± 0.13%, PRF: 73.76% ± 0.14%) [19], similar to the present study after three months. Alzahrani et al. measured significantly more socket filling with bone in the PRF group (88.8% ± 1.5%) compared to the control group (74.0% ± 1.2%) eight weeks post-extraction [16]. Temmerman et al. also found statistically significantly more socket filling in the PRF group (94.7% ± 26.9%) compared to the control group (63.3% ± 31.9%) after three months [26].

The percentage of socket filling with newly formed bone in the present study was comparable to the values reported in the studies listed. These values tended to be slightly higher, which may be due to the fact that most studies used only CBCT sections from the center of the socket or two-dimensional single-tooth radiographs, which did not account for the marginal areas of the socket. In these CBCT sections, the peripheral areas of the socket were not considered, and in the single-tooth radiographs, the potentially lower filling with regenerated bone in the peripheral areas was indistinguishable from the higher filling in the center. In the studies referenced here, except for Girish Kumar et al., the PRF groups showed significantly more socket filling with regenerated bone than the control groups [16,17,18,19,26]. In contrast, in the present study, only a tendency for more mineralized socket volume was observed in the solid PRF group, which could be due to the fact that the entire socket, including the peripheral areas with less filling, was assessed. It is also important to note that the comparability is limited due to the different time points of measurement after tooth extraction. For example, studies that measured socket filling with newly formed bone eight weeks post-extraction may have expected an increase in mineralization and a higher filling value at three months, which could not be detected in this study and may be somewhat misleading. A possible explanation for the lack of significant differences in mineralized socket volume between the solid PRF and control groups may lie in a revised understanding of alveolar healing dynamics. Recent insights suggest that post-extraction healing may not be driven primarily by uniform bone regeneration within the socket, but rather by a collapse of the alveolar walls, accompanied by appositional bone formation from the periphery. This could result in a seemingly well-mineralized crestal area while apical portions retain residual cavities [5]. Such a healing pattern might not be fully captured by conventional volumetric assessments, as it does not follow the typical endosteal bone growth seen in long bones. Since both groups described here rely on autologous blood-derived structures rather than mineralized bone substitute materials for extraction socket preservation—specifically, natural blood coagula in the case of spontaneous healing and solid PRF in the PRF group—their capacity to stabilize bone walls can be considered comparable. Therefore, further studies are needed to confirm this potential healing mechanism. In general, the mechanism proposed by Ghanaati et al. [5] should be taken into account when interpreting radiological data, particularly regarding the distribution of mineralized tissue within the socket. Given that the described method for volume and bone density measurement analyzes the entire socket, such changes will inevitably influence the measured outcomes—specifically, a reduction in bone volume due to collapsed walls and/or a decrease in the mean density of mineralized tissue due to residual non-mineralized cavities. Therefore, incorporating this method in future studies may provide valuable insights into the process of bone regeneration.

### 4.3. Efficacy of Solid PRF for Bone Regeneration—Radiological Bone Density

In addition to the newly formed mineralized tissue volume, the change in tissue density and degree of mineralization within the socket was assessed for the clinical trial. Three months after tooth extraction, the average bone density (measured from gray values) in the mineralized sockets showed no significant difference between the solid PRF and control groups, whether in molar or premolar sockets, or when subdivided into types 1 and 2.

The literature on radiologically determined bone density in extraction sockets with PRF application is limited, and methods for measuring bone density vary in accuracy. Sharma et al. found a significant increase in gray values, respective bone density, from immediately post-extraction to 16 weeks in both groups, but with no significant difference between them [28]. Srinivas et al. observed better radiological bone density in the PRF group at three months, though the data were not clearly evident [25]. Yewale et al. reported a significant increase in bone density in both groups from immediately post extraction to six months, with no significant difference between them [22].

The results of this study are only partially comparable to the literature due to differing approaches. Gray values, representing the bone or tissue density, in the cited studies are often reported as absolute values, while this study used relative values compared to the surrounding spongy bone, showing no significant difference between the PRF and control groups. Further clinical studies are needed to clarify whether PRF affects bone regeneration, potentially reflected in increased gray values within the socket. Future studies should use relative value reporting to ensure comparability, as described here.

### 4.4. Strength and Limitation of Radiological Evaluation

The creation of a macro code for Fiji to semi-automate the steps and guide users through the evaluation process, is making the method accessible to both novice and experienced users. Novices could apply the method without extensive training, while experienced users benefited from significantly faster analysis, especially during the marking and measuring stages. The reproducibility and transferability of the evaluation method were tested by having two users independently evaluate a set of CBCT images from the same study multiple times. The results demonstrated high consistency, with only minor deviations of less than 5%.

A notable limitation of the present and many other radiological methods is the constrained ability to infer the quality of newly formed tissue. Histological analyses, as recommended in the literature (e.g., Célio-Mariano et al. [35]), provide critical insights into tissue composition and mineralization. However, these analyses are invasive and can only sample limited portions of the socket. Therefore, combining histological and radiological methods is desirable and highly recommended to further enhance future study outcomes in regard to judging not just the quantitative, but also the qualitative aspect of the observed regeneration process.

The contextualization of results within the existing literature is complicated by the diverse methodological approaches employed. Approaches like that of Anitua et al. [34], which model the socket as a cone, are efficient in terms of time and resources but remain limited in their explanatory power. This study demonstrates that a detailed analysis encompassing the entire socket provides a more accurate depiction of bone regeneration. However, it must be noted that a standardized methodological framework within the literature is lacking, which significantly impedes the comparability of study results.

A fundamental limitation of radiological assessment methods in general, including the one developed in this study, is the assumption that socket healing primarily occurs through uniform bone regeneration within the extracted area [2]. However, emerging evidence suggests that alveolar healing may differ from classical endosteal bone regeneration and could instead involve a collapse of the socket walls with appositional bone deposition from the periphery [5]. This distinction is crucial, as conventional CBCT-based evaluations may not differentiate between true intraluminal bone formation and structural changes resulting from alveolar wall remodeling. Future research should integrate this perspective into image analysis methodologies, potentially incorporating additional parameters to distinguish between true bone regeneration and mechanical shifts in socket morphology.

## 5. Conclusions

The developed method offers higher precision compared to many approaches described in the literature, allowing for a more comprehensive assessment of bone regeneration. Even though that it is time-consuming and technically demanding, and its accuracy is dependent on the quality of the raw CBCT data. In contrast, many studies in the literature use more simplified, time-efficient methods, though they provide less detailed results. The future likely lies in combining radiological and histological methods, along with the development of semi-automated or automated segmentation techniques, which could enable more precise and time-efficient evaluations.

The first application of this method to a randomized controlled clinical trial evaluating solid PRF for socket preservation showed trends towards better mineralized bone volume and/or higher radiological bone tissue densities, although no statistically significant differences were detected in the evaluated cohort. Given the variety of methods and the need for clearer data, further clinical studies using the method described here—considering the entire socket—should assess both mineralized socket volume and radiological bone density as key parameters for evaluating bone regeneration with PRF application. Recent insights suggest that alveolar healing involves both alveolar wall remodeling and peripheral appositional bone deposition rather than uniform endosteal growth. Future studies should account for these structural changes to better distinguish true intraluminal bone regeneration from morphological adaptations of the alveolar ridge. This would provide more definitive insights into PRF’s efficacy in promoting bone regeneration, both alone and in combination with other biomaterials.

## Data Availability

Data are contained within the article.

## Abbreviations

The following abbreviations are used in this manuscript:

PRF	Platelet-rich fibrin
CBCT	Cone beam computer tomography
